# Complete Mitochondrial Genome Sequence of the Human Neuroblastoma Cell Line 751-NA

**DOI:** 10.1128/genomeA.01185-17

**Published:** 2017-11-16

**Authors:** Mao Zhang, Jian-Long Chen, Peng-Cheng Wang, Chuan-Yi Fu, Hao Peng

**Affiliations:** Department of Neurosurgery, Hainan General Hospital, Haikou, China

## Abstract

We report here for the first time the 16,566-bp mitochondrial genome sequence of the human neuroblastoma cell line 751-NA. The 13 protein-coding genes, 2 rRNAs, and 22 tRNAs are organized in a virtually identical fashion to a previously reported human mitochondrial genome, except that the 1,136-bp d-loop region is slightly variable between them.

## GENOME ANNOUNCEMENT

Mitochondrial DNA (mtDNA) is a closed, double-stranded, circular molecule that typically contains about 37 genes, including 2 rRNAs and 22 tRNAs. The instability and variation of mtDNA have been associated with many diseases in both animals and humans. Thus, the availability of a complete mtDNA sequence will assist in conducting clinical studies and developing new therapies for disease. The human neuroblastoma cell line 751-NA exhibits neuronal properties and a catecholaminergic phenotype (1). Treatment of the 751-NA cells with high concentrations of MPP+ for 3 days will produce cell death with clear morphological evidence of apoptosis (2).

We present here the complete 16,566-bp DNA sequence of the human neuroblastoma cell line 751-NA mitochondrial genome. PCR was carried out by a conventional procedure (3), and the genes were found to be organized in a fashion virtually identical to those found in a previously reported human mitochondrial genome (4). The genome included 13 protein-coding genes, 2 rRNAs, 22 tRNAs, and 1 control region. As a human genome, most protein-coding genes employed ATG as the start codon, while *ND2*, *ND3*, and *ND5* were initiated with ATA. The overall composition of the mitogenome was estimated to be 33.6% for A, 27.3% for T, 26.0% for C, and 13.1% for G with an A-T-rich (60.9%) feature. The *ND6* gene was encoded on the L strand, while the others were encoded on the H strand. These genes had four types of stop codons, including TAG for *ND2*, TAA for eight genes, AGA for *CytB*, and an incomplete termination codon T for *COX3*, *ND3*, and *ND4*. The lengths of the 12s rRNA gene and the 16s rRNA gene were 954 bp and 1,558 bp, respectively. They were located between the tRNA^Phe^ and tRNA^Leu^ genes and separated by the tRNA^Val^ gene. The putative origin of light-strand replication (OL), a small region, was located between tRNA^Asn^ and tRNA^Cys^, which corresponds to a length of 31 bp, and might be folded into a stable stem-loop secondary structure.

Like the pattern of slight-to-moderate sequence variations between the human neuroblastoma cell line 751-NA and human mitochondrial DNA found over most of the genome, the d-loop region of 1,136 bp in the human neuroblastoma cell line 751-NA is more variable than the human mitochondrial genome. This region is also quite variable in length and accounts for the size difference between the human mtDNA and the human neuroblastoma cell line 751-NA mtDNA.

### Accession number(s).

This mitochondrial genome sequence has been deposited at DDBJ/ENA/GenBank under the accession no. MF737176. The version described in this paper is the first version, MF737176.1.
